# Supplementary material for: TAQing2.0 for genome reorganization of asexual industrial yeasts by direct protein transfection
Source: Commun Biol. 2022 Feb 17;5:144. doi: 10.1038/s42003-022-03093-6 (PMC8854394; doi:10.1038/s42003-022-03093-6)
Supplement: Supplementary file 2 — Supplementary information [file 42003_2022_3093_MOESM2_ESM.pdf]

Supplementary information

TAQing2.0 for genome reorganization of asexual industrial yeasts by direct protein transfection

Taishi Yasukawa, *et al*.

Index:

Supplementary Figure 1 Cellular toxicity of the Xfect reagents. 2
Supplementary Figure 2 Comparison of images for estimating the level of background fluorescence or autofluorescence signals originated from live cells. 3
Supplementary Figure 3 Optimizing conditions for β-Gal transfection into yeast (Sc and Cu) intact cells by measuring β-Gal activities. 4
Supplementary Figure 4 Preparation and characterization of the 6×His-tagged NLS-TaqI. 6
Supplementary Figure 5 MA plots of RNA-seq experiments for AG4 and AG9 strains. 7
Supplementary Figure 6 An uncropped agarose gel image of pulse-field gel electrophoresis, source data for Fig. 3(e). 8
Supplementary Figure 7 A phylogenetic tree of Cu-related nonconventional/conventional yeasts. 9
Supplementary Table 1(a) Differential expression of flocculation genes in WT, AG4, and AG9. 10
Supplementary Table 1(b) Summary of raw data statistics in RNA-seq experiments. 10
Supplementary Table 2 Assembly statistics and Resequencing summary of WT Cu. 11
Supplementary Table 3 Chromosome information of WT reference genome. 11
Supplementary Table 4 List of primer sets used in this study. 12
Supplementary Table 5 Summary of SNVs in WT, TAQed mutant strains AG4 and AG9. 12
Supplementary Reference 13

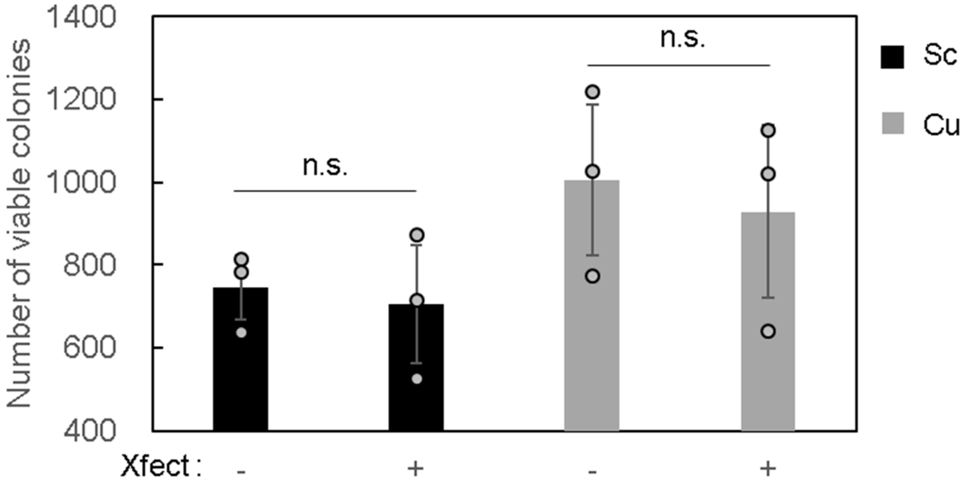

Supplementary Figure 1 Cellular toxicity of the Xfect reagents. Intact Sc and Cu cells suspended in PBS were mixed with (+) or without (-) Xfect at 30°C for 120 min according to the procedure in the methods section. The cells were then cultured on YPD agar plates at 30ºC for a few days, and formed colonies were counted. The bars and error bars respectively represent the mean and standard deviation from three independent experiments (*n* = 3). n.s. means not significant.

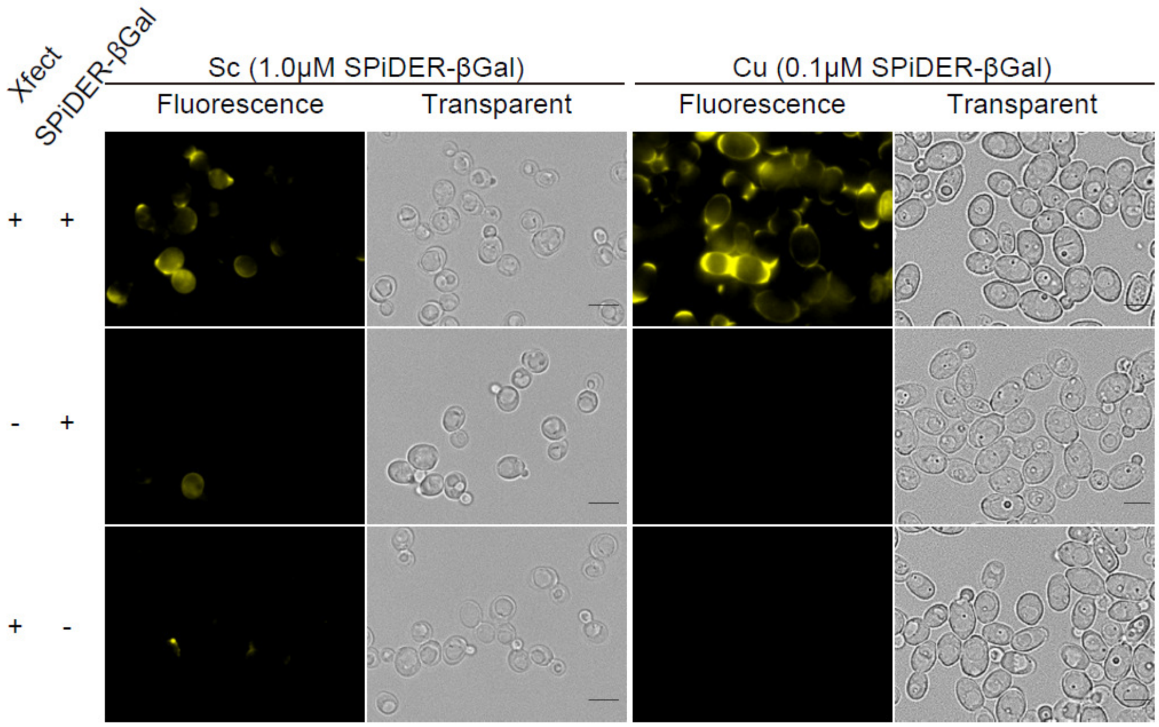

Supplementary Figure 2 Comparison of images for estimating the level of background fluorescence or autofluorescence signals originated from live cells. In the same number of Sc as Cu, intact cells were mixed with β-Gal with (+) or without (-) Xfect at 30°C for 60 min according to the standard protocol. After removal of extra β-Gal or Xfect/β-Gal by washing cells, an aliquot of cells was suspended with SPiDER-βGal (1 μM for Sc; 0.1 μM for Cu). All images were acquired as described in Fig. 1**(c)**. Scale bar = 5 μm.

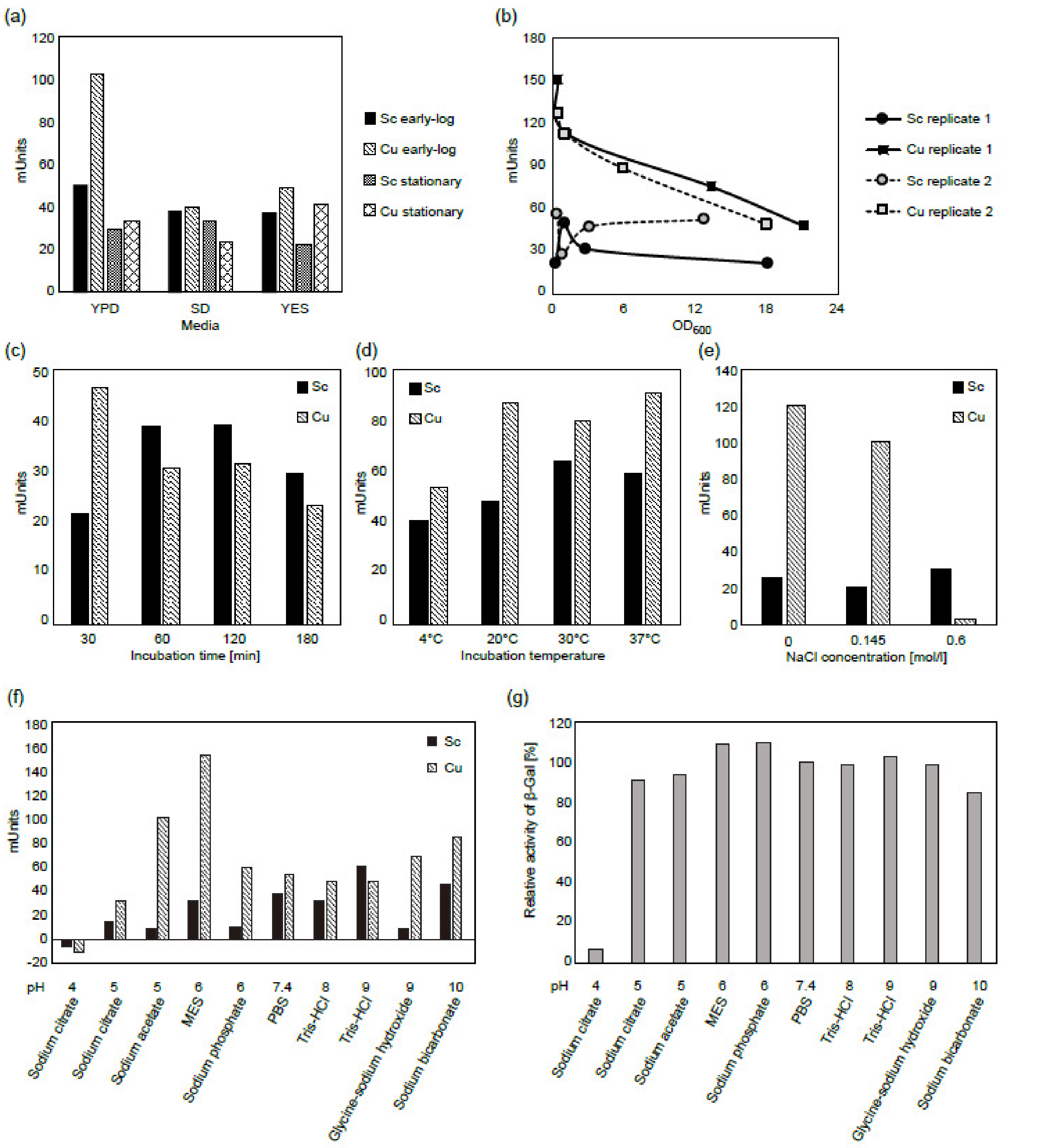

Supplementary Figure 3 Optimizing conditions for β-Gal transfection into yeast (Sc and Cu) intact cells by measuring β-Gal activities. Two categories, namely, cell cultivating conditions **(a, b)** and protein transfection conditions **(c-g),** were examined for β-Gal transfection efficiency into yeast cells. The β-Gal activity is shown as mUnits except for (g). **(a)** Effects of media for cultivating yeast cells and growth phases on β-Gal transfection. Yeast cells grown in the indicated medium and harvested at the indicated phase were subjected to β-Gal transfection, and the β-Gal activity was measured as described in Materials and Methods. **(b)** Effects of yeast growth phases on β-Gal transfection. Yeast cells harvested at the indicated OD_{600} were analyzed as above. **(c)** Effects of Xfect incubation time on β-Gal transfection. Yeast cells were

treated with β-Gal/Xfect complexes in PBS buffer (pH 7.4) at 30°C for 30, 60, 120, or 180 min, and the β-Gal activity was measured as above. **(d)** Effects of Xfect incubation temperature on β-Gal transfection. Yeast cells were treated with β-Gal/Xfect complexes in PBS buffer (pH 7.4) for 120 min at 4, 20, 30, or 37°C, and the β-Gal activity was measured as above. **(e)** Effects of NaCl concentration on β-Gal transfection. β-Gal/Xfect complexes were mixed with yeast cells in HEPES (pH 7.4) with 0, 0.145 or 0.6 mol/l NaCl, and the β-Gal activity was measured as above. **(f)** Effects of pH and buffer on β-Gal transfection. Yeast cells were transfected with β-Gal in the multiple pH/buffer conditions, and the β-Gal activity was measured according to the colorimetric assay described in Materials and Methods. **(g)** Effects of pH and buffer on β-Gal activity. The activity of commercially available β-Galactosidase (TaKaRa Bio Inc., Shiga, Japan) was examined under several pH and buffer conditions tested in (f). The relative β-Gal activities, where the activity in PBS (pH 7.4) was set to 100%, are shown.

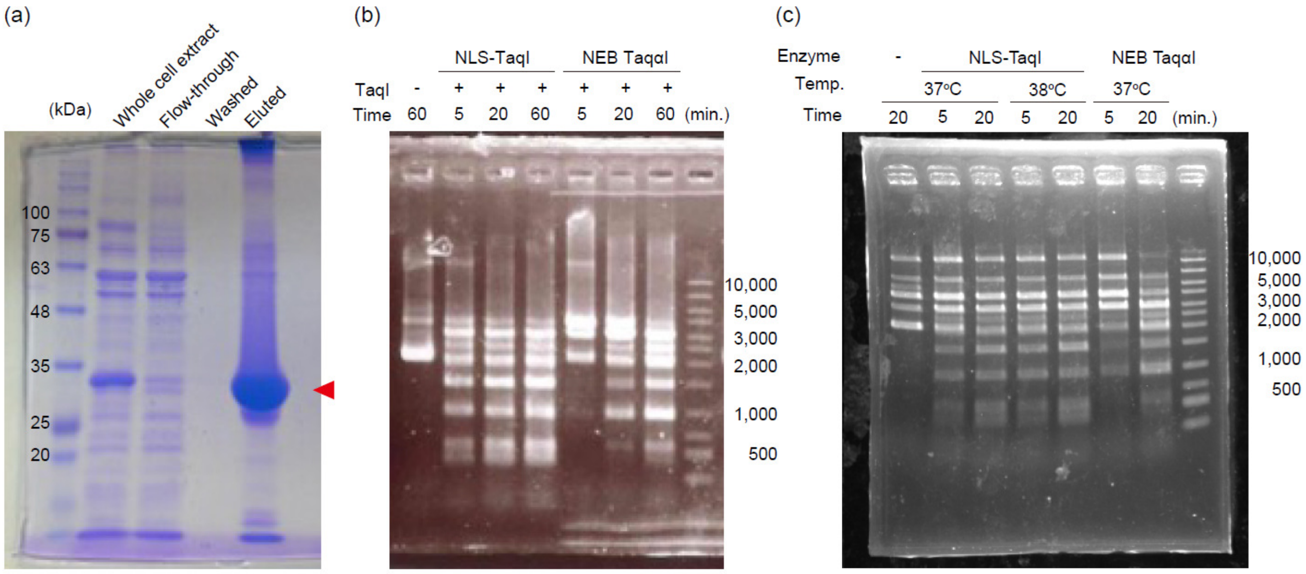

Supplementary Figure 4 Preparation and characterization of the 6×His-tagged NLS-TaqI. (a) An SDS-PAGE profile of purified 6×His-tagged NLS-TaqI. Whole cell extract (Lane 1), flow-through fraction (lane 2), washed fraction (Lane 3), and eluted fraction (Lane 4) were analyzed by SDS-PAGE and Coomassie Brilliant Blue staining. **(b)** Validation of enzyme/substrate specificity of the 6×His-tagged NLS-TaqI. 6×His-tagged NLS-TaqI purified as in (a) along with commercially available Taqαl (New England BioLabs) were incubated with the substrate pBluescript II SK(+) for the indicated time (min) at 37°C, and the DNA was analyzed by agarose-gel electrophoresis and ethidium bromide staining. **(c)** Comparison of NLS-TaqI activity at 37°C and 38°C. 6xHis-tagged NLS-TaqI was incubated with the pBluescript II SK(+) for the indicated time (min) at 37°C or 38°C. For comparison, Taqαl was treated at 37°C for the indicated time (min).

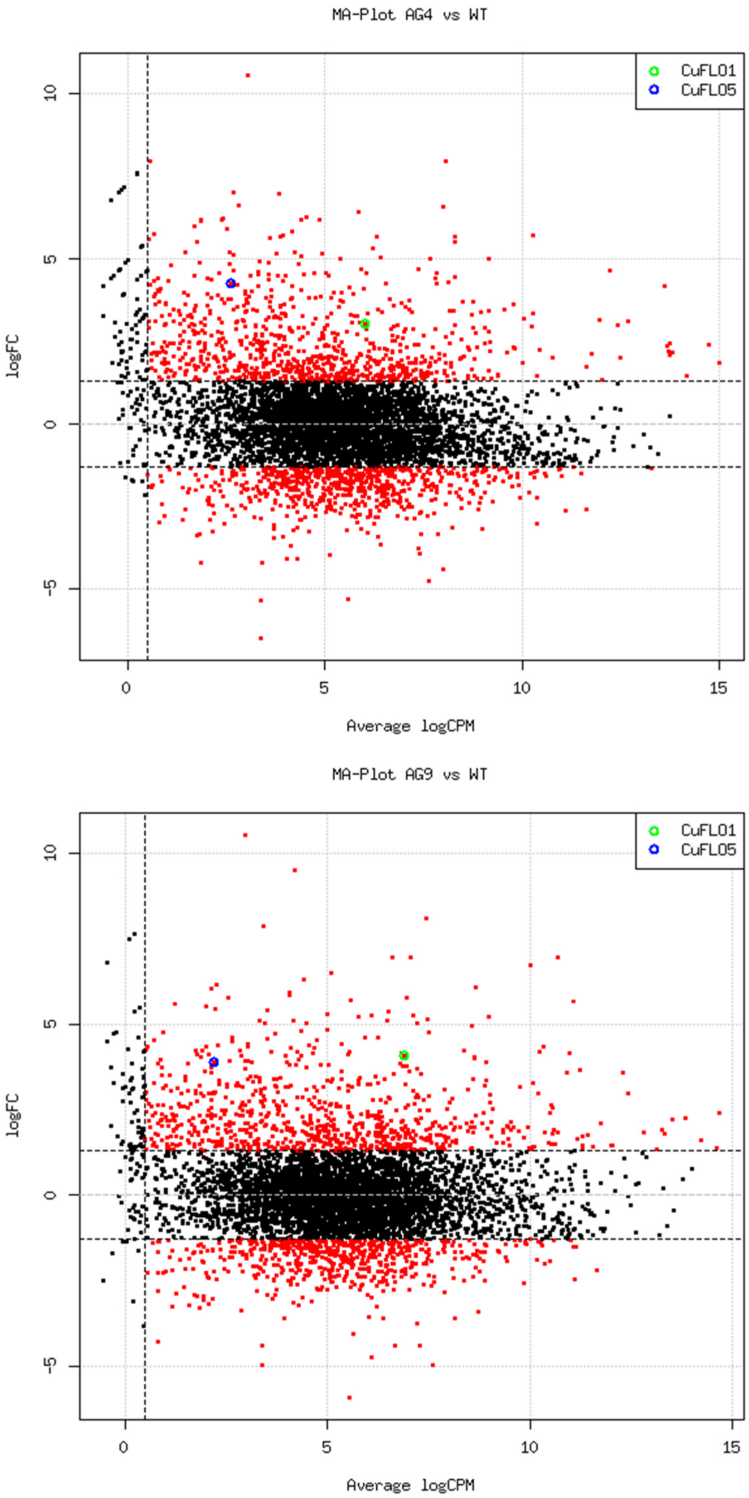

Supplementary Figure 5 MA plots of RNA-seq experiments for AG4 and AG9 strains. MA-plots of genome-wide gene expression data, AG4/WT (upper), AG9/WT (lower). Red dots indicate genes whose average logCPMs are larger than 0.5 and the absolute values of Log_2FC are larger than 1.3. The green dots and the blue dots indicate *CuFLO1* and *CuFLO5* gene, respectively.

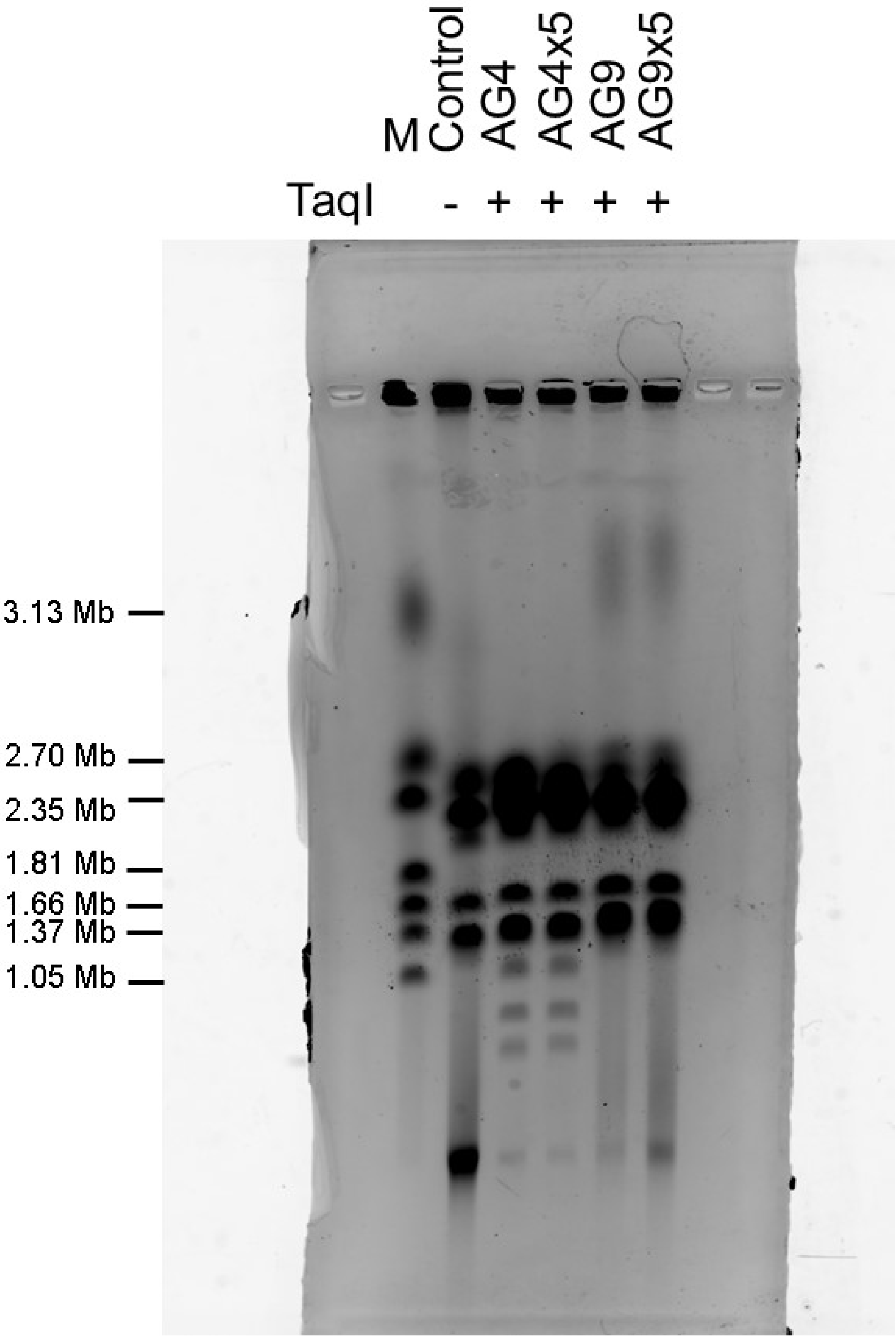

Supplementary Figure 6 An uncropped agarose gel image of pulse-field gel electrophoresis, source data for Fig. 3(e).

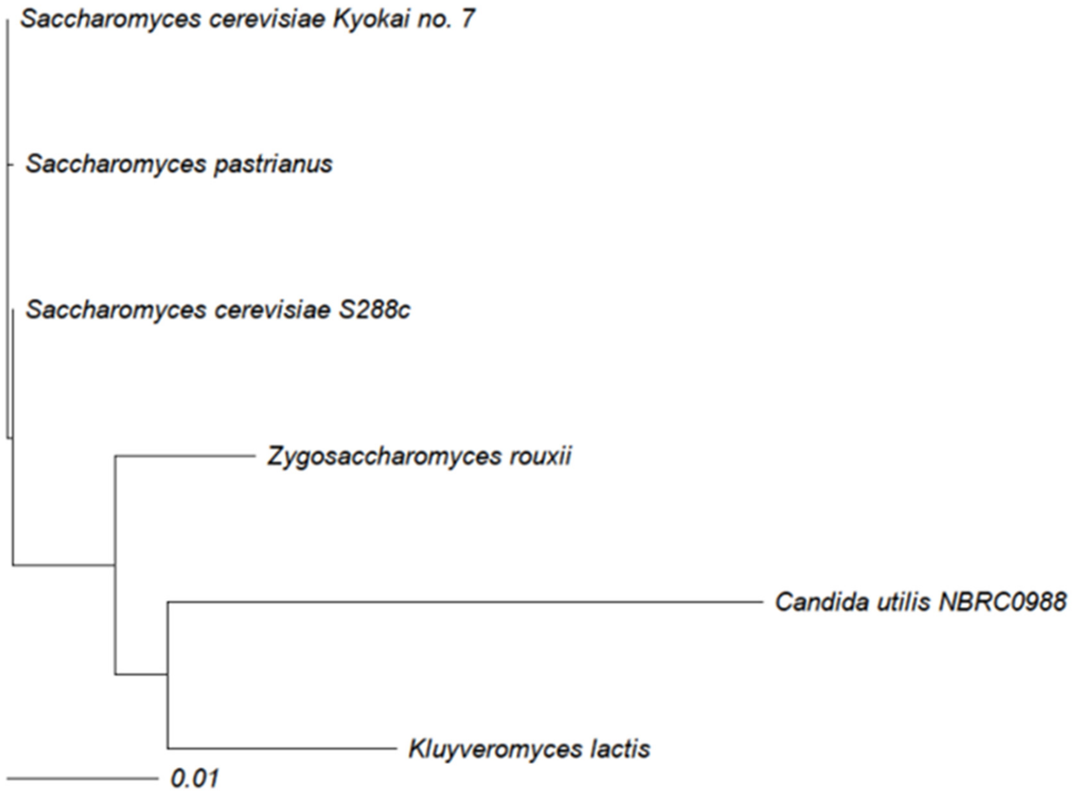

Supplementary Figure 7 A phylogenetic tree of Cu-related nonconventional/conventional yeasts. The phylogenetic analysis was based on comparison with their 18S rRNA gene sequences (about 1,800 bp), which were obtained by this study (*C. utilis* NBRC0988) or from RNAcentral database [1] (https://rnacentral.org/)). *S. cerevisiae* S288c (URS00005F2C2D_559292), *S. cerevisiae* Kyokai no. 7 (URS00005F2C2D_721032), *S. pastrianus* (URS00008CAE4A_27292), *Zygosaccharomyces rouxii* (URS00003FC14D_4956), *Kluyveromyces lactis* (URS000080E249_28985). Multiple sequence alignments were calculated using DDBJ ClustalW (ver. 2.1, https://clustalw.ddbj.nig.ac.jp/). The phylogenetic trees were drawn by using TreeViewX program (ver. 0.5.0). The scale bar represents substitution ratio of base among sequences.

Supplementary Table 1(a) Differential expression of flocculation genes in WT, AG4, and AG9. Expression changes of some flocculation-related genes and housekeeping genes (*ACT1*, *RPL3*) are listed.

	Cu ID	Amino acid sequence similarity to Sc counterparts	AG4 / WT		AG9 / WT	
	evm.model.		Log_2FC	fold changes	Log_2FC	fold changes
FLO1	ChrV.357	little	3.05	8.3	4.07	17
FLO5	ChrV.499<br>ChrIII.223	42.9%<br>33.2%	1.12<br>4.25	2.2<br>19	0.81<br>3.90	1.8<br>15
FLO8	ChrI.1188	44.0%	-0.97	0.51	-0.57	0.67
FLO9	-	little	-	-	-	-
FLO10	-	little	-	-	-	-
FLO11	ChrV.545	31.0%	0.12	1.1	-0.19	0.88
ACT1	ChrIII.214	95.2%	-0.15	0.90	0.30	1.2
RPL3	ChrII.429	88.9%	-0.92	0.53	-0.98	0.51

Supplementary Table 1(b) Summary of raw data statistics in RNA-seq experiments.

Sample ID	Total read bases (bp)	Total reads	GC (%)	AT (%)	Q20 (%)	Q30 (%)
CuWT_1	4,750,597,410	31,460,910	47.68	52.32	97.35	93.54
CuWT_2	5,638,261,178	37,339,478	47.75	52.25	97.27	93.36
CuAG4x5_1	5,850,157,062	38,742,762	48.45	51.55	97.48	93.81
CuAG4x5_2	5,493,346,780	36,379,780	48.39	51.61	97.35	93.51
CuAG9x5_1	6,163,762,922	40,819,622	48.24	51.76	97.06	92.95
CuAG9x5_2	4,675,448,334	30,963,234	48.20	51.80	97.42	93.67

Supplementary Table 2 Assembly statistics and Resequencing summary of WT Cu.

	FALCON			MaSuRCA
	Primary contigs + Haplotigs	Primary contigs	Haplotigs	
Contigs	263	29	234	30
Total length	30,802,437	13,271,634	17,530,803	14,097,946
Contig N50	893,192	1,375,218	469,640	1,821,643
Largest contig	1,914,286	1,914,286	1,333,929	2,138,172
Average length	117,120	457,643	74,918	469,932
G+C contents	44.59%	44.51%	44.64%	44.42%

Supplementary Table 3 Chromosome information of WT reference genome.

	Chromosome length (bp)
ChrI	4,541,153
ChrII	2,424,584
ChrIII	2,119,607
ChrIV	1,514,913
ChrV	1,274,270
ChrVI	1,241,925

Supplementary Table 4 List of primer sets used in this study.

Names in Fig.4(d)	Sequences 5' → 3'	Product size (bp)	Note
F	AGATAAGGAGTAAGAATTATGATCTCTACA	700	
R	ACTGCTATAGTCTGTGAGTAGTTAAA		

Names in Fig. 4(e)	Sequences 5' → 3'	Product size (bp)	Note
1	CTAACAAAAGTTATGGGTCAGTATT		Universal for WT and AG4 without SNVs
2	CTA**G**CAAAAGTTATGGG**C**CAGTAT**C**		Bold letters: AG4 strain specific SNVs
3	AAGACGTTACCGATAAGGAAATA	2,000	For the TL detection
4	GAAGATCAAAGTTTCCAATGAAGAA	1,800	For an inversion detection
4'	TTCTTCATTGGAAACTTTGATCTTC		
5	TGGAAATTGCATTGAGATACTTATTC		
5'	GAATAAGTATCTCAATGCAATTTCCA	2,100	For the TL detection

In Methods	Sequences 5' → 3'	Product Size (bp)	Note
	CATATGTGTACTCCACCTAAAAAGAAG<br>CGTAAAGTTGCCCCTACACAAGCCCA<br>GGATCCTTACGGGCCGGTGAGGGC	831	TaqI-coding DNA fragment with NLS tag at N-terminus

Supplementary Table 5 Summary of SNVs in WT, TAQed mutant strains AG4 and AG9.

	WT	AG4	AG9
SNVs	229,216	191,237	205,774
InDels	16,428	13,670	14,686

Supplementary Reference

[1] The RNAcentral consortium. RNAcentral: a hub of information for non-coding RNA sequences. *Nucleic Acids Res.* **47,** 221-229 (2019).
