# Supplementary material for: TAQing2.0 for genome reorganization of asexual industrial yeasts by direct protein transfection
Source: Commun Biol. 2022 Feb 17;5:144. doi: 10.1038/s42003-022-03093-6 (PMC8854394; doi:10.1038/s42003-022-03093-6)
Supplement: Supplementary file 3 — Description of Additional Supplementary Files [file 42003_2022_3093_MOESM3_ESM.pdf]

Description of Additional Supplementary Files

File name: Supplementary Data
Description: Source data for underlying the graphs and plots in the main figures.
