# Supplementary material for: TAQing2.0 for genome reorganization of asexual industrial yeasts by direct protein transfection
Source: Commun Biol. 2022 Feb 17;5:144. doi: 10.1038/s42003-022-03093-6 (PMC8854394; doi:10.1038/s42003-022-03093-6)
Supplement: Supplementary file 5 — Reporting Summary [file 42003_2022_3093_MOESM5_ESM.pdf]

nature portfolio

Corresponding author(s): Kunihiro Ohta

Last updated by author(s): Jan 24, 2022

Reporting Summary

Nature Portfolio wishes to improve the reproducibility of the work that we publish. This form provides structure for consistency and transparency in reporting. For further information on Nature Portfolio policies, see our Editorial Policies and the Editorial Policy Checklist.

Statistics

For all statistical analyses, confirm that the following items are present in the figure legend, table legend, main text, or Methods section.

n/a	Confirmed	
☐	☒	The exact sample size (n) for each experimental group/condition, given as a discrete number and unit of measurement
☐	☒	A statement on whether measurements were taken from distinct samples or whether the same sample was measured repeatedly
☐	☒	The statistical test(s) used AND whether they are one- or two-sided<br>*Only common tests should be described solely by name; describe more complex techniques in the Methods section.*
☒	☐	A description of all covariates tested
☒	☐	A description of any assumptions or corrections, such as tests of normality and adjustment for multiple comparisons
☐	☒	A full description of the statistical parameters including central tendency (e.g. means) or other basic estimates (e.g. regression coefficient) AND variation (e.g. standard deviation) or associated estimates of uncertainty (e.g. confidence intervals)
☐	☒	For null hypothesis testing, the test statistic (e.g. F, t, r) with confidence intervals, effect sizes, degrees of freedom and P value noted<br>*Give P values as exact values whenever suitable.*
☒	☐	For Bayesian analysis, information on the choice of priors and Markov chain Monte Carlo settings
☒	☐	For hierarchical and complex designs, identification of the appropriate level for tests and full reporting of outcomes
☒	☐	Estimates of effect sizes (e.g. Cohen's d, Pearson's r), indicating how they were calculated

Our web collection on statistics for biologists contains articles on many of the points above.

Software and code

Policy information about availability of computer code

Data collection	We used PacBio Sequel, Illumina MiSeq, Illumina NovaSeq, and Illumina platform, based on Illumina sequencing by synthesis (SBS) technology. Microscopic images were acquired by the BZ-X700 system (Keyence).
Data analysis	The raw sequencing data processing, de novo genome assembly of Candida utilis (Cu), resequencing and RNA-seq analysis of Cu mutants, obtained by the TAQing2.0 system, were performed using available tools as described in the main manuscript. One-tailed Welch's t-tests were performed using Microsoft Excel. The fluorescence intensity from microscopic images was measured and analyzed using the hybrid cell-counting tool BZ-H3C (Keyence).

For manuscripts utilizing custom algorithms or software that are central to the research but not yet described in published literature, software must be made available to editors and reviewers. We strongly encourage code deposition in a community repository (e.g. GitHub). See the Nature Portfolio guidelines for submitting code & software for further information.

Data

Policy information about availability of data

All manuscripts must include a data availability statement. This statement should provide the following information, where applicable:

- Accession codes, unique identifiers, or web links for publicly available datasets
- A description of any restrictions on data availability
- For clinical datasets or third party data, please ensure that the statement adheres to our policy

Sequencing data are deposited in the DDBJ/EMBL/GenBank database under accession numbers AP024664-AP024669 and DDBJ Sequence Read Archive database under accession number DRA012057, DRA012846, and DRA012997. Gene expression data of RNA-seq experiments are deposited in DDBJ Genomic Expression Archive (GEA) under accession number E-GEAD-459. Source data for underlying the graphs and plots in the main figures are provided in Supplementary Data. An

uncropped agarose gel image of pulse-field gel electrophoresis (Fig. 3(e)) is in Supplemetary Figure 6. All other data are available from the corresponding authors upon reasonable request.

Field-specific reporting

Please select the one below that is the best fit for your research. If you are not sure, read the appropriate sections before making your selection.

☒ Life sciences ☐ Behavioural & social sciences ☐ Ecological, evolutionary & environmental sciences

For a reference copy of the document with all sections, see nature.com/documents/nr-reporting-summary-flat.pdf

Life sciences study design

All studies must disclose on these points even when the disclosure is negative.

Sample size	At least three biological replicates were performed in the colorimetric and yeast viability experiments, where we performed in triplicates per each biological replicate. All microscopic images were acquired 3–10 fields for each replicate, and each quantitative experiment was analyzed using more than triplicate fields; the exact number of sample size is described in the main manuscript.
Data exclusions	No data were excluded from analyses.
Replication	Reproducibility was confirmed by performing at least two independent experiments.
Randomization	Microscopic images were randomly selected in the fluorescent-based quantitative measurement between yeast cells.
Blinding	No blind experiments.

Reporting for specific materials, systems and methods

We require information from authors about some types of materials, experimental systems and methods used in many studies. Here, indicate whether each material, system or method listed is relevant to your study. If you are not sure if a list item applies to your research, read the appropriate section before selecting a response.

Materials & experimental systems

n/a	Involved in the study	
☐	☒	Antibodies
☐	☒	Eukaryotic cell lines
☒	☐	Palaeontology and archaeology
☒	☐	Animals and other organisms
☒	☐	Human research participants
☒	☐	Clinical data
☒	☐	Dual use research of concern

Methods

n/a	Involved in the study	
☒	☐	ChIP-seq
☒	☐	Flow cytometry
☒	☐	MRI-based neuroimaging

Antibodies

Antibodies used	Alexa Fluor 488 goat anti-rabbit IgG (Cell Signaling Technology, product No. 4412S)
Validation	Affinity purified antibodies.

Eukaryotic cell lines

Policy information about cell lines

Cell line source(s)	Candida utilis NBRC0988<br>Saccharomyces cerevisiae S288c
Authentication	We purchased Candida utilis strain from the National Institute of Technology and Evaluation, and Saccharomyces cerevisiae strain from Summit Pharmaceuticals International Corporation, authorized distributors of authentic ATCC.
Mycoplasma contamination	Not tested for mycoplasma contamination.
Commonly misidentified lines (See ICLAC register)	Not relevant.
